# Supplementary material for: Synthesis of Superheat-Resistant Polyimides with Enhanced Dielectric Constant by Introduction of Cu(ΙΙ)-Coordination
Source: Polymers (Basel). 2020 Feb 13;12(2):442. doi: 10.3390/polym12020442 (PMC7077670; doi:10.3390/polym12020442)
Supplement: Supplementary file 1 [file polymers-12-00442-s001.pdf]

Electronic Supplementary Information (ESI):

Synthesis of Superheat-Resistant Polyimides with Enhanced Dielectric Constant by Introduction of Cu(II)-Coordination

Guangtao Qian, Mengjie Hu, Shangying Zhang, Mengxia Wang, Chunhai Chen and Jianan Yao *

Center for Advanced Low-Dimension Materials, State Key Laboratory for Modification of Chemical Fibers and Polymer Materials, College of Material Science and Engineering, Donghua University, Shanghai 201620, China; 1179133@mail.dhu.edu.cn (G.Q.); 2180373@mail.dhu.edu.cn (M.H.); 1185099@mail.dhu.edu.cn (S.Z.); 2180257@mail.edu.edu.cn (M.W.); cch@dhu.edu.cn (C.C.)

* Correspondence: yjn@dhu.edu.cn; Tel.: +86-021-67798670

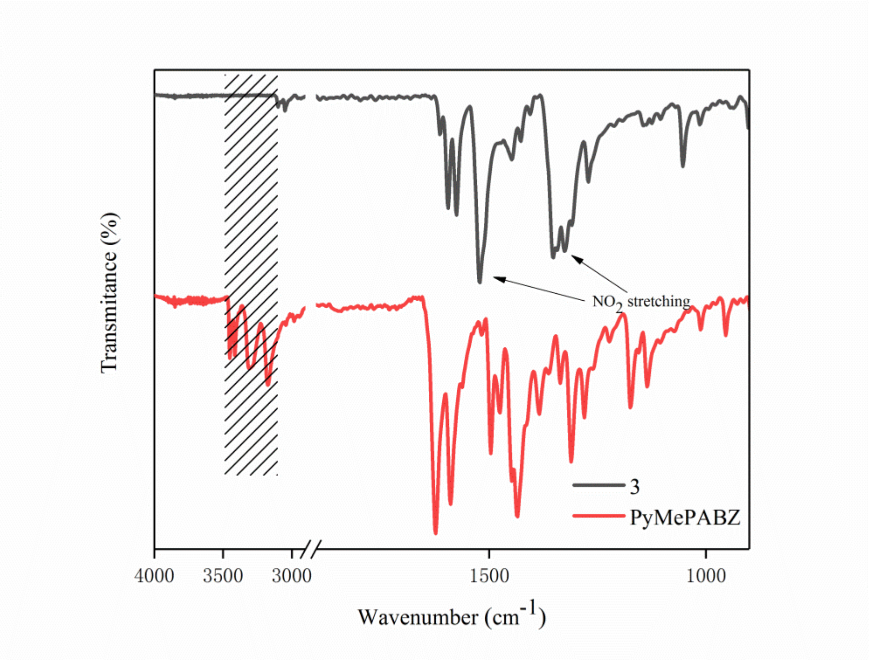

Figure S1. IR spectra of 3 and PyMePABZ.

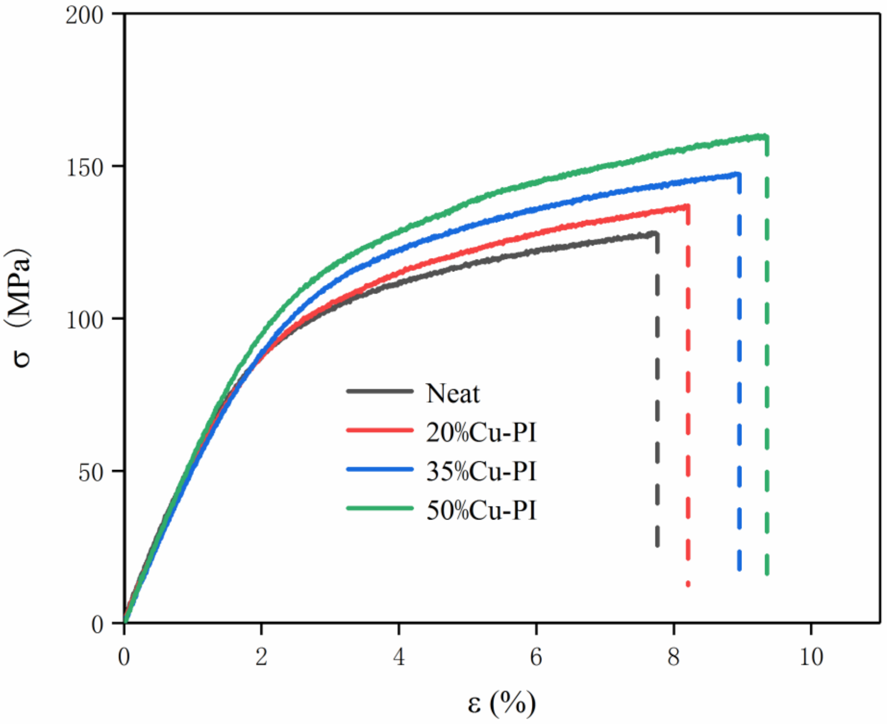

Figure S2. Stress–strain curves of neat PI and Cu-PI films.
